# Editorial and Miscellaneous

**Published:** 1875-12

**Authors:** 


### Editorial and Miscellaneous.

*The Old and New.*

TEMPUS FUGIT was said centuries ago with as slight appreciation, by the multitude, of what the phrase really means, as in our day, when the ancient axiom is sentimentally repeated in men by the trite phrase, "Time flies." And yet this same time, which we are prone to contemplate only in the abstract, or metaphorically, or as a rhetorical ornament, a winged image of the imagination, or portrayed by pen, and pencil, as a reaper with death-dealing scythe—this fancy-invested "time," we say, is nevertheless the most real, solemn and sublimely-impressive element of human life.

Time is visible Eternity; the "tick, tick" of the clock is the audible pulse-throb of Creation; the passing hours are tokens and emblems of the Everlasting: they come and go, and go and come, with the incessant regularity impressed upon them by the Divine Will, as the physical expression of its own unceasing harmony; they are the comprehensible parts of a cycle, within whose circumference all that was, is, or can be, is encircled, and which contains all that is conceivable or inconceivable of the material and spirtual creations of Jehovah.

Viewed in this aspect, and it is the only one in which this subject should be viewed by creatures endowed with the faculty of reason, and the fruits of knowledge, time is entitled to all the considerations we have given it, and, at once, becomes one of the vastest themes that human thought can consider.

Such contemplation becomes especially appropriate at the period when the year—that rapidly decreasing measure of our earth-existence—is near its close, and the heart is awakened to the fact that a new year, with all its untried hopes and fears, is standing at our threshold, ready to assume the falling sceptre of its predecessor. At such a time the soul, as though it were vivified by the magical touch of some angelic presence, rises to a higher level, and endeavors to pierce the misty horizon of the future with longing eyes; and Memory, grasping her pilgrim staff, begins to retrace the darkening path behind her—why? There are lost jewels to be recovered, which in the heat and turmoil of life's battle dropped unnoticed from the heart; but she remembers them now, and wishes them reset in the heart's crown. There are graves of dead hopes to be visited and adorned, for love's sake, with lilies and forget-me-nots; there are pleasant bowers to be sought, to which once Life turned aside for rest and solace; where the fragrance of roses from the garden of Joy scented the summer airs—where the golden harmonies of Peace lulled the over-burdened spirit to repose, and celestial dreams came down from the star-lands, to abide a moment with the weary wanderer.

Through all this region of the past, over which the sun-set of the year flings its weird glamour, Memory wanders, to stop at last at a tomb over which rank grasses grow, and where the spirit of neglect holds his dismal court: it is the Tomb of Lost Opportunities. Oh! could we recall them to life! They blossomed all along the pathway of the year, like flowers in the wake of the rejoicing spring. In their chalices shone the wine of gladness; in their leaves lay the honey-dew of contentment; their roots were nurtured by celestial juices; they were blessed by ministering angels, but we knew it not; we passed them by as if they were weeds unworthy of our notice, and the little golden opportunity, which might have made us happy, and good, and strong in soul, and worthier of God's love, and more useful to our fellow-men, drooped of neglect, and died, and its dust is now laid forever in yonder desolate tomb, where Memory kneels to weep her useless tears.

Ah! when the new year has passed over our threshold and begins to record our lives in the great book of Life, will we profit by the experience gained in the old? Will we endeavor to make ourselves better, purer, nobler and worthier for the glorious work which the Allwise Father in Heaven has given us to do? Will we embrace the golden opportunities of fleeting time, instead of neglecting them, to our loss and sorrow? Will we put forth all our strength of mind in the pursuit of useful knowledge, and in the achievement of all that is best in science, art and human progress, in order that the world may be benefitted by our labor, and made better because of our having lived in it? In substance, will we be what we ought to be, and can be, if we will?

Let us not delay, then, to lay the lessons of the past year to our hearts, and let us imbue our souls with the wisdom of experience, that the new year may blossom as the rose, and be a fragrant, acceptable offering, to be laid before the Lord of Love and Grace when the new year, too, shall have waxed old, and is gathered to the dust of the dead past—forever.

Now, as to the RECORD and its management during the past year, its conductor desires to say that if he has done anything during that period which was calculated to wound the sensibility of any one, he begs forgiveness for it, assuring all that any dereliction on his part was unintentional. During the coming year he will, more strenuously than ever, guard against any slip of this nature, hoping that the subscribers of the RECORD will resolve to do the same, in the spirit of the "Golden Rule."

The outstanding dues for subscription are coming in rapidly, accompanied by the kindest of letters, assuring us that the delay in paying the subscription was purely negligence on the writer's part, and expressing regrets at having caused us inconvenience or loss by the delay. Such appreciation of our labors and expressions of sympathy touches our heart, and makes us rejoice to know that we have not been mistaken in placing a high estimate upon the character of our patrons. The half dozen who misunderstood our urgent appeal for remittance of subscription, and answered us tartly, have doubtless, ere this, regretted their hastiness, and we assure them that there is no ill will against them in our heart.

If these friends will renew their subscriptions to the RECORD, we assure them they will have cause for congratulation. We again urge upon our friends to work for the RECORD. We ask each one to secure for us one new subscriber for 1876. P.

*Our Associate Editors and Co-Laborers.*

We would be neglectful of our duty did we omit to mention the valuable assistance which we have received from our corps of associate editors and co-laborers, in the work of conducting the RECORD. We sincerely thank them for this work during the past year, and trust they will continue to enrich these pages by their contributions. Much of the high reputation enjoyed by the RECORD in the world of medical literature is due to the effectual labor and talent of our numerous corps of assistants, and we take great pleasure in making the statement. Let their diligence and zeal, for the welfare of the RECORD, be doubled during the coming year; the field is ample and the harvest sure for the faithful laborer.

*To Our Exchanges.*

The pleasant relationship which the Record has held with its numerous and valuable exchanges, during the past year, is a matter especially worthy of note. In extending to our brethren of the press, most heartily, the compliments of the season, the Editors of the Record hope that the fraternal feelings of the past will be continued, without interruption, through the coming year, and that encouragement and pleasure will result from the mutual efforts of the "noble fraternity of quill drivers" to build up the temple of knowledge, and to assist the progress, and ensure the welfare, of mankind.

